# The effects of maternal dietary fiber and short-chain fatty acids on the health of offspring: a mini review

**DOI:** 10.3389/fnut.2026.1867014

**Published:** 2026-09-03

**Authors:** Shaozhi Liu, Senling Feng, Zhongwen Yuan, Zhengrong Mei, Jinjin Yin, Lei Chen, Yongxia Zhang

**Affiliations:** 1 Guangdong Provincial Key Laboratory of Major Obstetric Diseases, Department of Pharmacy, Guangdong Provincial Clinical Research Center for Obstetrics and Gynecology, The Third Affiliated Hospital, Guangzhou Medical University, Guangzhou, China; 2 Guangdong Provincial Key Laboratory of Major Obstetric Diseases, Department of Cardiovascular Medicine, Guangdong Provincial Clinical Research Center for Obstetrics and Gynecology, The Third Affiliated Hospital of Guangzhou Medical University, Guangzhou, China

**Keywords:** dietary fiber, gut microbiota, maternal nutrition, metabolic programming, offspring health, short-chain fatty acids

## Abstract

Maternal nutrition during pregnancy is a critical determinant of offspring long-term health. Dietary fiber has emerged as a key modifiable factor. Through gut microbial fermentation, fiber is converted into short-chain fatty acids (SCFAs), primarily acetate, propionate, and butyrate. This mini review synthesizes current evidence from molecular, animal, and human studies on the role of maternal SCFAs in programming offspring metabolic and immune health. Mechanistically, SCFAs act via two complementary pathways: activation of G protein-coupled receptors (GPCRs) and inhibition of histone deacetylases, leading to rapid signaling and long-term epigenetic regulation. Animal studies demonstrate that maternal fiber deprivation impairs offspring glucose metabolism, promotes low-grade inflammation and obesity, and compromises immune tolerance, whereas fiber or short-chain fatty acid (SCFA) supplementation confers protective effects. Human observational studies support these findings, showing associations between maternal SCFA levels and favorable neonatal metabolic profiles, neurodevelopmental outcomes, and reduced obesity risk, although randomized controlled trials remain limited. Key translational challenges include inter-species differences, individual microbiome variability, and optimal intervention timing. Despite these uncertainties, promoting adequate and diverse dietary fiber intake during pregnancy represents a safe, low-cost, and scalable public health strategy to improve intergenerational health outcomes.

## Graphical abstract

**Figure:**
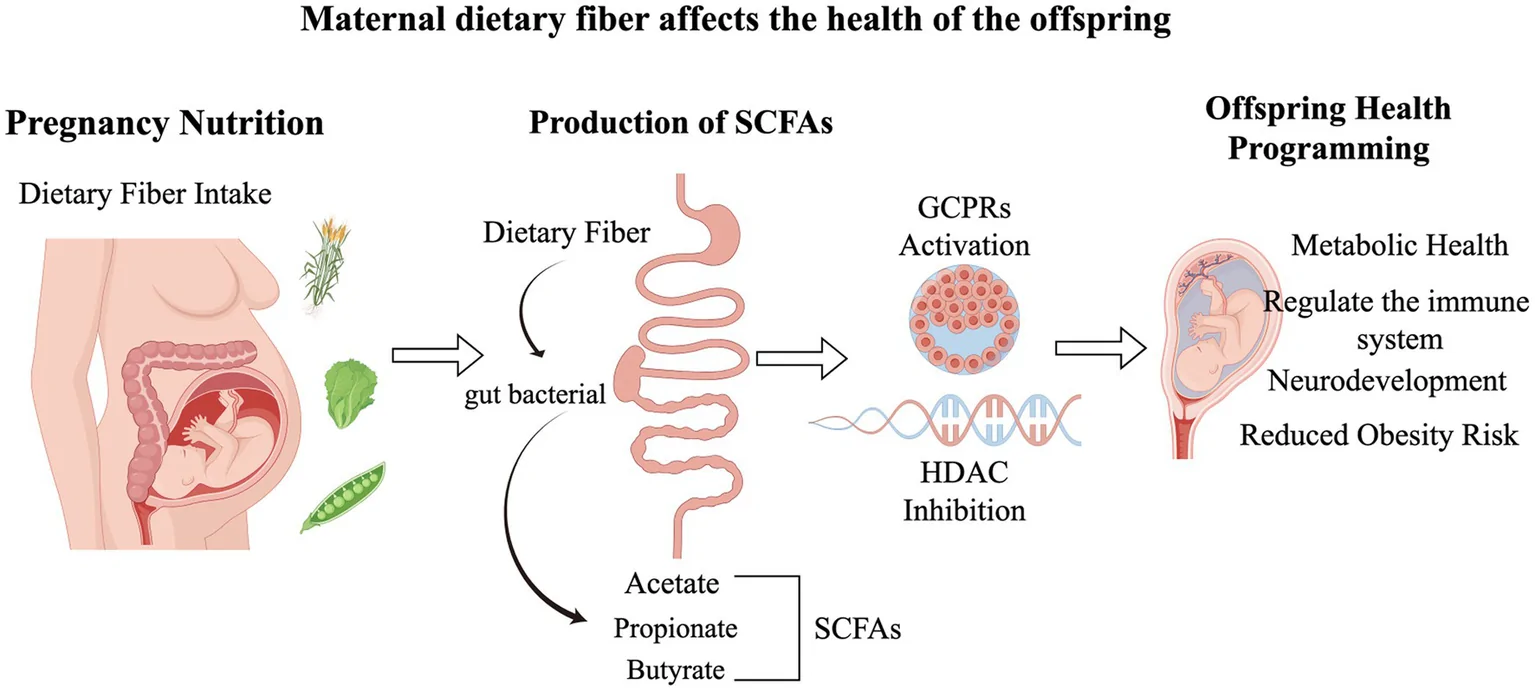
Created with Figdraw.com.

## 1 Introduction

Non-communicable diseases, encompassing obesity, type 2 diabetes mellitus and cardiovascular disease, represent one of the most pressing public health challenges of the twenty-first century, imposing an escalating and unsustainable burden on healthcare systems and economies worldwide (1, 2). Genetic predisposition and adult lifestyle behaviors remain important determinants of disease risk. However, the developmental-origins hypothesis has fundamentally transformed our etiological understanding, this hypothesis demonstrates that susceptibility to disease is frequently programmed during the earliest and most biologically plastic stages of human development (3, 4). The intrauterine period constitutes a critically sensitive window during which the fetus continuously adapts its physiological architecture in response to signals from the maternal environment, a process termed developmental programming (5).

The intrauterine milieu is shaped by maternal nutritional status, body composition, metabolic health, and psychosocial stress, each contributing to the long-term calibration of offspring physiological function (6, 7). Beyond its conventional role in digestion, the maternal gut microbiome functions as a dynamic, diet-responsive endocrine organ that ferments otherwise inaccessible dietary substrates, generating bioactive metabolites capable of traversing the placental barrier to directly interface with the developing fetus (8–11). Among these microbial-derived signals, SCFAs, including principally acetate, propionate, and butyrate, generated through anaerobic fermentation of dietary fiber have emerged as potent molecular mediators with broad developmental programming capabilities (12, 13).

The present review provides a comprehensive mechanistic synthesis of how maternal dietary fiber intake, through SCFA production by the gut microbiota, programs the metabolic and immune development of offspring. This synthesis integrates evidence from molecular pathways, animal experiments, and human studies, while examining the translational challenges that must be addressed to convert these insights into clinical interventions.

## 2 Metabolism and function of SCFAs

### 2.1 Composition and classification of SCFAs

SCFAs are primarily synthesized through the gut bacterial fermentation of dietary fiber. SCFAs are fatty acids with carboxylate anions containing fewer than six carbon atoms. This group includes acetic acid (C2:0), propionic acid (C3:0) and butyric acid (C4:0) (14). In humans, the most abundant SCFAs are acetic, propionic, and butyric acids, which are typically found in a consistent ratio of 3:1:1 in colon (14). The concentration of SCFA varies by gastrointestinal site: the proximal colon has a higher concentration, while the distal colon has a lower concentration, which is related to the increased supply of carbohydrates and water in the proximal colon (15). Bacterial fermentation acts on non-digestible carbohydrates. These include plant cell wall polysaccharides, resistant starch, and soluble oligosaccharides, all of which are found in dietary fibers (16). Dietary fiber can be divided into two forms: soluble and insoluble. Soluble fibers include pectin, inulin, psyllium husk, etc., while insoluble fibers include cellulose, hemicellulose, etc. (17). Resistant starch serves as a substrate specifically for butyrate synthesis, while pectin and inulin contribute mainly to the production of acetate and propionate (17). Additionally, certain prebiotics such as inulin, fructo-oligosaccharides (FOS), and galacto-oligosaccharides (GOS) have been shown to boost SCFAs production (17).

### 2.2 Production and *in vivo* distribution of SCFAs

Different intestinal bacteria produce different amounts of SCFAs (18). Gram-negative bacteria such as Bacteroides mainly produce acetate and propionate, whereas Gram-positive bacteria such as Firmicutes mainly produce butyrate (18). The proximal colon has the most vigorous growth, reproduction, fermentation, and activity of microorganisms due to its rich supply of nutrients, while the cecum and proximal colon are the main sites of SCFA production, and the distal colon contributes the least due to the least supply of nutrients (15).

Once synthesized within the intestinal lumen, SCFAs undergo rapid absorption and utilization by colonocytes. Butyrate functions as the principal oxidative fuel for these epithelial cells, with the vast majority being consumed locally at the colonic mucosa (19, 20). Acetate and propionate, by comparison, are transported to a considerably greater degree into the portal venous system, subsequently reaching hepatic and systemic tissues (19, 20). Propionate is metabolized predominantly in the liver, where it serves as a precursor for gluconeogenesis and thereby contributes to the maintenance of glucose balance (19, 20). Acetate, upon entering the systemic circulation, is distributed to multiple organ systems, including adipose depots, skeletal muscle, and the central nervous system, where it participates in the modulation of whole-body energy homeostasis (19, 20). As a result, insufficient consumption of dietary fiber diminishes SCFA output, with downstream consequences for host physiological function (21).

### 2.3 Mechanisms of action of SCFAs

SCFAs, as the primary metabolites of dietary fiber fermentation by the gut microbiota, exert a wide range of biological functions through two highly synergistic core mechanisms: activation of GPCRs and inhibition of histone deacetylases (HDACs). The former mediates rapid signal transduction, while the latter enables long-term epigenetic regulation of gene expression. Together, these mechanisms achieve functional complementarity across multiple tissue types.

#### 2.3.1 Receptor-mediated signaling of SCFAs

Among the approximately 800 GPCRs in the human genome, a cluster of four GPCR genes (GPR40 to GPR43) is located in close proximity to the CD22 gene on chromosome 19q13.1 (20). These receptors are named free fatty acid receptors (FFARs) because they sense free fatty acids (20). In 2003, GPR43 and GPR41 were deorphanized by three independent research groups and subsequently renamed FFAR2 and FFAR3, respectively (20). At the receptor level, SCFAs serve as endogenous ligands for GPR41, GPR43, and G protein-coupled receptor 109A (GPR109A) (22–24). They bind to these receptor subtypes with differential affinities (24), GPR41 shows a higher affinity for acetate and propionate, the three ligands activate GPR43 with similar efficacy, and only butyrate activates GPR109a (14, 25, 26). GPR43 is highly expressed in enteroendocrine L cells, colonic Treg cells, pancreatic islets, a portion of immune cells, and adipocytes (27–29). The transcription of human GPR43 and mouse GPR43 has also been found to be expressed in neutrophils and eosinophils, mediating chemotaxis and anti-inflammatory effects (30). GPR41 is highly expressed in sympathetic ganglia and has a direct regulatory effect on sympathetic nerve activity. Moreover, GPR41 has been reported to be expressed in adipose tissue and promotes the secretion of leptin (31). Immunohistochemistry for GPR41 showed that mucosal GPR41 protein is localized in cytoplasm of enterocytes and enteroendocrine cells (32). SCFAs impact the host’s metabolism by promoting the release of glucagon-like peptide-1 (GLP-1) and peptide YY (PYY), thereby regulating insulin secretion, intestinal motility, and central appetite control (20). The impact on intestinal immunity is mainly achieved through the secretion of more interleukin-18 (IL-18) by intestinal epithelial cells and through the butyrate stimulation signal transduction of GPR109A, which leads to the generation of regulatory T cells (Tregs) and T cells that produce interleukin-10 (IL-10) (33)_._ In mice, forkhead box P3 (Foxp3) expression is essential for the regulatory function of CD25^+^ T cells (34). Tregs are a specific subset of T cells, possessing the potential to inhibit and maintain the inflammatory homeostasis (35). In pregnant mice, butyric acid reduces NF-κB signal transduction (36). Additionally, it was associated with increased body weight gain and elevated levels of blood glucose, insulin, triacylglycerol, and cholesterol (36). Voltolini et al. (37) demonstrated the expression of GPR41/GPR43 in human placental and fetal membrane tissues. Propionic acid reduced the chemotactic activity of neutrophils and the protein secretion of neutrophil chemotactic factor interleukin-8 (IL-8) induced by lipopolysaccharide (LPS) (37). The inhibition of the inflammatory pathway by SCFA may have therapeutic benefits for pregnant women at risk of preterm birth due to pathogenic factors (37). In mice, propionate that reaches the fetal bloodstream has been shown to play a dual role: it regulates insulin levels via GPR43 signaling and influences sympathetic nervous system development through GPR41 signaling (38). The presence of GPR41 and GPR43 receptors in the uteroplacental unit, along with their involvement in inflammatory processes during pregnancy and delivery, may represent a key pathway through which SCFAs influence fetal metabolic programming (39–41). SCFA has extensive effects on the host: metabolism, differentiation, and proliferation, mainly due to their influence on gene regulation.

#### 2.3.2 Epigenetic regulation of SCFAs

Among the SCFAs, butyrate has been the most extensively studied. Numerous studies have shown that butyrate regulates 5 to 20% of human gene expression (14). At the cellular level, both butyric acid and propionic acid inhibit lysine and histone deacetylase (K/HDAC) activity, with butyric acid being more potent than propionic acid (14). *In vitro* HDAC inhibition assay revealed that SCFAs inhibit HDAC3 more potently than HDAC7, with butyric acid being the most potent HDAC3 inhibitor among SCFAs (IC_50_ = 0.318 mM) (42). At the epigenetic level, butyrate acts as an endogenous HDAC inhibitor, modulating gene expression by increasing histone acetylation levels and promoting an open chromatin structure (3, 43–45). Representative examples include enhancing acetylation of the Foxp3 gene promoter to facilitate Treg lineage commitment (46), and regulating genes involved in hepatic gluconeogenesis and lipid metabolism to achieve long-term programming of metabolic homeostasis (26, 47). Inhibition of HDAC by SCFAs modulates immune responses to commensal organisms. SCFA also regulates the expression of cytokines in T cells and the generation of Tregs by inhibiting HDAC. Effect T cells (T_H_1, T_H_2, and T_H_17 cells) have enhanced aerobic glycolysis, and the inhibition of glycolysis promotes the generation of Treg cells (48).

**Figure 1:**
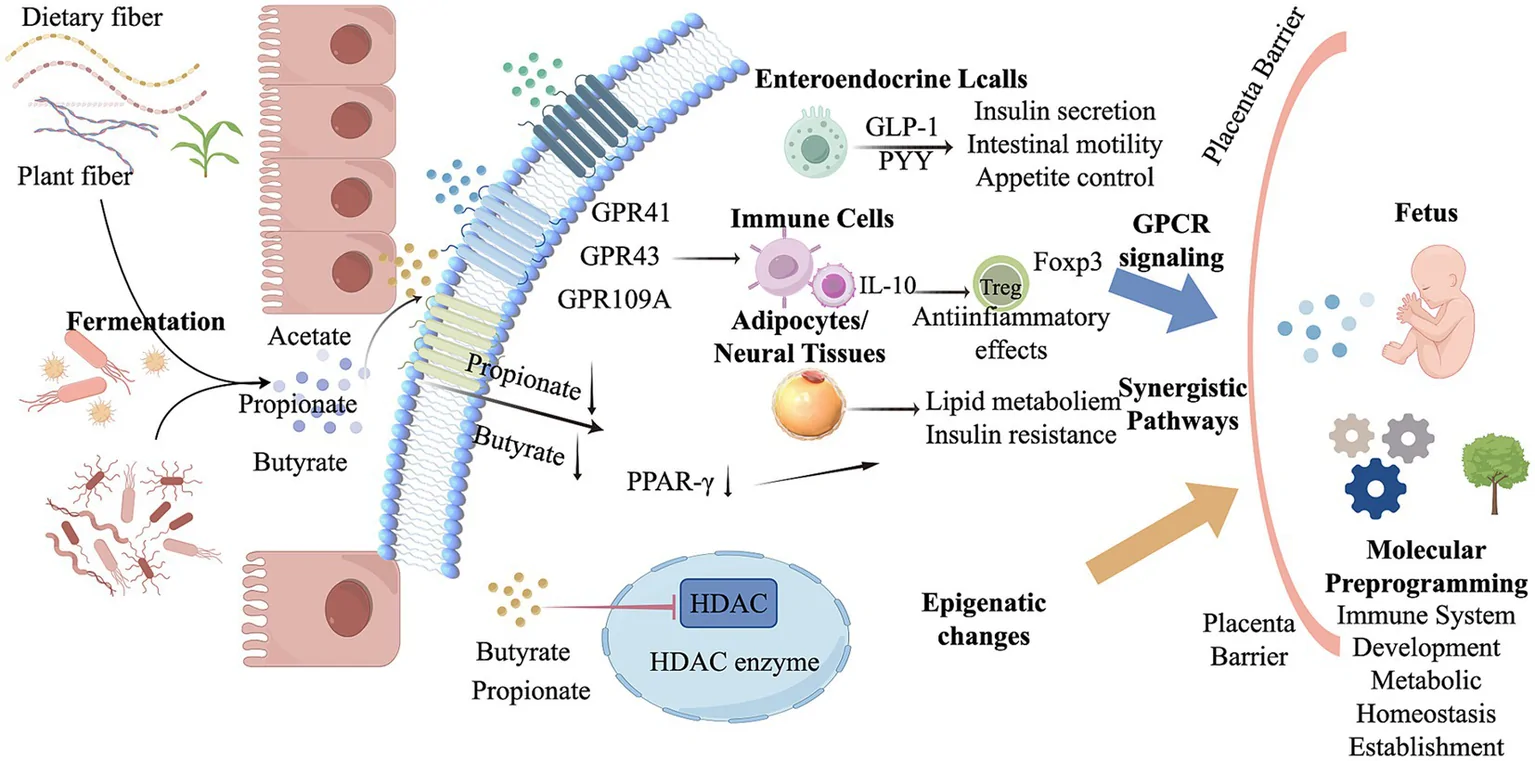
Dual mechanisms of short-chain fatty acids (SCFAs) in maternal-fetal programming. SCFAs are the primary metabolites produced by gut microbiota through dietary fiber fermentation. They influence offspring health via two highly synergistic core mechanisms, namely the activation of G protein-coupled receptors (GPCRs) and the inhibition of histone deacetylases (HDACs). GPCR, G protein-coupled receptor; GPR41/43/109A, G protein-coupled receptor 41/43/109A; PPAR-γ, peroxisome proliferator-activated receptor γ; HDAC, histone deacetylase; GLP-1, glucagon-like peptide-1; PYY, peptide YY; IL-10, interleukin-10; Treg, regulatory T cell; Foxp3, forkhead box P3. Created with Figdraw.com.

Furthermore, peroxisome proliferator-activated receptor PPAR, as one of the important and effective molecules downstream of SCFAs, is a group of nuclear transcription factors that regulate gene expression (25). The PPAR nuclear receptor family consists of three isotypes: *α*, *β* and *γ*, PPAR-β and PPAR-γ have been demonstrated to play important roles in placental formation, with PPAR-γ in particular thought to be crucial for the progression of pregnancy (49). During the first trimester of human pregnancy, PPAR-*γ*is mainly expressed in villous cytotrophoblasts and invading extravillous trophoblasts, with this pattern observable as early as 7 weeks of gestation (49, 50). In the second trimester, PPAR-γ is present in cytotrophoblasts as well as in the columnar structures of the anchoring villi. In the third trimester, PPAR-*γ*expression in the human placenta is found in villous syncytiotrophoblasts, along with both villous and extravillous cytotrophoblasts (49). SCFAs may promote the transition from lipid synthesis to lipid utilization involves the targeting of PPAR-γ, this leads to increased expression of mitochondrial uncoupling protein 2 and an elevated AMP to ATP ratio, which in turn activates AMPK and stimulates oxidative metabolism in the liver and adipose tissue (51). Animal experiments have shown that when the concentration of SCFA decreases, the expression of HDAC3 increases, H327K acetylation in the intestine decreases, resulting in the inhibition of PPAR-*γ*, and further upregulate CD36, thereby causing excessive fatty acids and triglycerides to be transported into the bloodstream, leading to insulin resistance (52). Butyric acid is one of the known SCFAs that can activate PPAR-*γ* (52). Butyrate, as an endogenous PPAR-*γ* agonist, directly binds to PPAR-*γ*, promoting the transcriptional activity mediated by PPAR*γ*, thereby increasing the number of Treg cells in visceral adipose tissue (53). Furthermore, butyrate was further shown to promote, similarly to PPAR-*γ* ligands, the interactions between PPAR-γ and multiple coactivators, and to bind into the PPAR-γ binding pocket with a conformation resembling that of the known PPAR-γ agonist, decanoic acid (54). However, the specific mechanisms by which SCFAs regulate PPAR function during pregnancy remain to be fully elucidated. The mechanism diagram is shown in Figure 1.

### 2.4 Placental transfer and milk secretion of short-chain fatty acids

SCFAs produced by gut microbial fermentation are absorbed by intestinal epithelial cells and enter the maternal circulation (25). Although low-level microbial colonization may exist in the intrauterine environment, SCFA concentrations in the maternal circulation are substantially higher than those in the fetus, suggesting that the mother serves as the primary source of fetal SCFAs (55, 56). Animal studies have further shown that maternal dietary fiber intake during pregnancy significantly increases SCFA concentrations in the amniotic fluid, indicating that the fetus is indeed exposed to maternal-derived SCFAs in utero (57, 58).

Mechanistically, SCFAs can be transported across cell membranes in their ionized form via monocarboxylate transporters (MCTs) and sodium-coupled MCTs (SMCTs) (25). MCTs act as mediators for the transport of SCFAs. So far, 14 members of MCT have been identified but only MCT1-4 have been expressed in an active form and characterized as proton-linked MCT1 was found in almost all tissues (59). The placenta expresses SCFA-related transporters (MCT1 and MCT4) (60, 61), MCT1 and MCT4 were, respectively, localized on the basal membrane and the apical microvilli membrane of human placental syncytiotrophoblast cells (62). Moreover, MCT1 and MCT4 are highly expressed during the early stage of pregnancy, indicating that SCFAs are actively transported during the early stage of embryonic development. MCT1 and MCT4 show polarized distribution on the microvillus membrane (MVM) and basal membrane (BM) sides of the syncytial growth layer (STB) (25). MCT1 is the main transporter for transporting acetic acid, and MCT1 functions on both the apical and basal membranes of the syncytiotrophoblast (63). As well as receptors such as FFARs, including GPR41 and GPR43 (25, 57, 58). These molecular features indicate that SCFAs may be transported across the placenta into the fetal circulation and exert receptor-mediated effects on fetal development and metabolic reprogramming. Nevertheless, the specific regulatory mechanisms of placental FFARs remain to be elucidated.

In addition to transplacental transfer, SCFAs are also delivered to neonates through breast milk during lactation. SCFAs generated by the maternal gut microbiota are presumed to reach the mammary gland via the systemic circulation and be subsequently secreted into breast milk (64). Previous studies have detected SCFAs such as acetate, propionate, and butyrate in human breast milk (65, 66), and Maternal dietary fiber intake modulates SCFA production through gut microbial fermentation, with implications for both maternal metabolic health and offspring development. In the mother, SCFAs such as butyrate and propionate help attenuate adipose tissue inflammation and improve insulin sensitivity, which is particularly relevant in the context of gestational diabetes mellitus (GDM) (58, 67). In parallel, maternal microbial metabolites, including SCFAs, can shape the offspring’s immune development by promoting Treg cells expansion and supporting mucosal immune maturation (58, 67).

Collectively, these findings indicate that maternal gut microbial metabolites can continuously influence early offspring development through two distinct routes: transplacental transfer during gestation and lactational transmission via breast milk.

### 2.5 Maternal intestinal barrier

Compared with non-pregnant women, the levels of SCFAs in pregnant women during the early stage of pregnancy increase (25). As the pregnancy progresses, the diversity of vaginal or colonic microbiota gradually decreases, which is consistent with the trend of a reduction in total SCFAs (25). Changes in gut microbiota metabolites during pregnancy have immune-regulatory effects that help maintain the balance of the gut immune system, thereby influencing intestinal permeability (68). Intestinal homeostasis is of vital importance to the host’s health, and the intestinal barrier is crucial for preventing the invasion of bacteria and toxins, thereby supporting intestinal health and the normal functioning of the immune system (69). SCFAs, as the main metabolic products of gut microbiota from fermenting dietary fibers, can enhance the intestinal epithelial barrier function and reduce the translocation of LPS into the bloodstream (70). Among them, butyric acid strengthens the intestinal barrier through multiple mechanisms. At the protein assembly level, butyric acid can activate AMP-activated protein kinase (AMPK), promoting the redistribution and assembly of tight junction proteins. This process does not rely on significant changes in the expression level of tight junction proteins, although the upstream activation mechanism remains to be clarified (71). At the transcriptional regulation level, butyric acid promotes the specific binding interaction between transcription factor SP1 and the specific binding sites of the Claudin-1 promoter region, thereby upregulating the transcriptional expression of Claudin-1 (72). These two pathways complement each other and jointly maintain the integrity of the intestinal epithelial barrier.

When the intestinal barrier function is impaired, bacterial-derived LPS can translocate through the intestinal wall into the systemic circulation, inducing metabolic endotoxemia and systemic low-grade inflammatory response. In the obese mouse model, prebiotic intervention can selectively regulate the structure of the intestinal microbiota, and improve intestinal permeability through a GLP-2-dependent mechanism, reducing portal LPS levels, and thereby alleviating systemic inflammation and metabolic disorders (73).

### 2.6 Cross-regulatory network of SCFAs and other microbial metabolites

SCFAs should not be interpreted in isolation but rather situated within a broader microbial metabolite network. In pregnancy, their production and biological effects are intertwined with other gut-derived metabolites through substrate competition, microbial ecological interactions, and convergent host signaling pathways (20). The relationship between bile acids and the gut microbiota is bidirectional. Microbial enzymes generate secondary bile acids that modulate farnesoid X receptor and the G protein-coupled membrane receptor 5 signaling, while bile acids themselves selectively reshape microbial community structure and fermentative capacity (74). Indole metabolites derived from tryptophan activate aromatic hydrocarbon receptor (AhR) to reinforce mucosal immune homeostasis (75, 76), while butyrate enhances epithelial barrier function through GPR109A activation and HDAC inhibition (77). Both pathways converge on barrier protection and anti-inflammatory regulation at the mucosal interface. When dietary fiber is insufficient, fermentation substrates shift toward proteins, reducing SCFA generation while increasing branched chain fatty acids and other proteolytic metabolites (78). SCFAs also counteract metabolic endotoxemia by upregulating tight junction proteins and suppressing NF-κB-mediated inflammation (77, 79). Together, these findings suggest that the effect of maternal dietary fiber depends not only on SCFA levels themselves, but also on the broader landscape of microbial metabolites, which collectively shape maternal metabolism and influence immune-metabolic programming in offspring.

## 3 Maternal SCFA signaling and fetal developmental programming

Maternal gut microbiota-derived SCFAs, particularly propionic acid, cross the placenta and are secreted into breast milk to shape fetal developmental programming. Kimura et al. (38) showed in germ-free and low-fiber diet mouse models that maternal propionic acid activates sympathetic ganglia, intestine, and pancreas in embryos, promoting differentiation of sympathetic neurons, GLP-1 enteroendocrine cells, and pancreatic *β* cells, thereby establishing postnatal energy homeostasis. Offspring of germ-free or low-fiber diet mothers developed worsened obesity and glucose intolerance on a high-fat diet, which was reversed by propionic acid supplementation during pregnancy (38). In contrast, Zou et al. (80) found that maternal fiber deficiency during lactation leads to persistent alterations in the offspring’s gut microbiota, characterized by reduced microbial diversity and increased abundance of proteobacteria. Even when the offspring were fed a fiber-rich diet for a long period after weaning, this microbial dysbiosis persisted, accompanied by increased bacterial invasion of the intestinal mucus layer and low-grade chronic inflammation. As a result, these offspring were more susceptible to obesity and insulin resistance when fed a high-fat diet in adulthood (80). Sanna et al. (81) further confirmed through Mendelian randomization studies that the increase in intestinal butyrate production driven by host genetics is significantly correlated with the improvement in insulin secretion response in the oral glucose tolerance test, while the genetic prediction of elevated fecal propionic acid levels has a causal relationship with an increased risk of type 2 diabetes.

The liver is the core organ for metabolism. Different SCFAs have differential regulatory effects on its metabolic functions. Studies have shown that both acetic acid and butyric acid are negatively correlated with impaired glucose tolerance, suggesting that they may have potential protective effects on glucose metabolism (82). Among them, butyric acid was also negatively correlated with gestational weight gain and systemic inflammatory levels (82). Acetic acid has been proven to reduce the accumulation of triglycerides, thereby improving lipid metabolism (83). The mechanism of butyric acid is more complex. It can inhibit appetite through the vagus nerve pathway, activate brown adipose tissue, increase energy consumption by inhibiting HDAC, and improve insulin sensitivity at the same time (84, 85). These mechanistic studies have provided an important foundation for understanding the regulatory role of SCFAs in the host’s energy metabolism. In clinical studies, the levels of SCFAs during the maternal pregnancy period are closely related to various metabolic indicators and neonatal outcomes. The research shows that the maternal serum acetic acid level is positively correlated with gestational weight gain and adiponectin levels, while propionic acid is negatively correlated with leptin levels, neonatal height and weight (86). Furthermore, the levels of maternal SCFAs are also significantly correlated with indicators such as the body fat distribution of the newborns (25, 86–88). These findings suggest that maternal SCFAs may influence the metabolic state of the mother and the growth and development of the fetus by regulating the levels of adipokines such as adiponectin and leptin.

In terms of neurodevelopment, prospective cohort studies have shown that adequate dietary fiber supplementation during pregnancy is associated with infant neurodevelopment (89). Prenatal depression symptoms can cause dysbiosis of the maternal gut microbiota during pregnancy, which in turn affects the neurodevelopment of the offspring. 16S rRNA sequencing revealed that the levels of butyric acid-producing bacteria were lower in patients with prenatal depression (90). These characteristics were associated with poorer cognitive outcomes in the offspring (90). Another prospective study based on the FinnBrain Birth Cohort indicates that the composition of the infant’s gut microbiota is associated with their emotional state at 6 months of age (91). More importantly, the cognitive and social functional impairments in offspring caused by maternal obesity and high-fat diet exposure can be improved through high-fiber dietary intervention. Recent studies have found that maternal obesity can cause cognitive and social behavioral deficits in human and mouse offspring (92). However, maternal or offspring intake of high-fiber diets can improve these behavioral abnormalities (92). This improvement is achieved by reshaping the gut microbiota and restoring the levels of SCFAs such as acetic acid and propionic acid in feces (92). From a mechanistic perspective, supplementing SCFAs can significantly upregulate the expression of SCFAs receptors Olfr78 in the hippocampus and prefrontal cortex of offspring, and by regulating the expression of genes related to microglial cell maturation and function (such as PSD-95, etc.), it can improve the length and width of the postsynaptic dense zone (PSD), thereby protecting the synaptic structure of the hippocampus and prefrontal cortex (92).

In terms of immune function, a high-fiber diet during the maternal pregnancy and lactation period can increase the levels of SCFAs in the offspring and increase the frequency of thymic Tregs and peripheral Foxp3 Tregs (93). Mechanistic studies have shown that butyric acid can, through a GPR41 receptor-dependent pathway, upregulate the expression of autoimmunomodulatory factors in the epithelial cells of the thymic medulla, thereby promoting the differentiation of tTregs and the establishment of central immune tolerance (93). In the maternal inflammation model, butyric acid and propionic acid were able to inhibit the mRNA expression and secretion of pro-inflammatory cytokines and chemokines induced by TNF or LPS in placental tissues, demonstrating anti-inflammatory effects (94). Maternal gestational diabetes and obesity can alter the composition of the early colonizing bacterial community in the offspring (41). The study found that the reduction of butyric acid-producing and propionic acid-producing bacteria in the early stages of life, along with a decrease in fecal SCFAs levels, is associated with an increased risk of atopic eczema and allergen sensitization in the offspring (95). Those whose maternal feces contained a relatively high level of acetic acid had offspring with a lower risk of developing atopic asthma or wheezing (96). Another cohort study showed that children with the highest levels of butyric and propionic acids in their feces at the age of one had significantly reduced atopic allergic reactions, and were less likely to develop asthma between the ages of 3 and 6 (97). Regulatory T lymphocytes generate a tolerant immune profile and promote the Th1 phenotype to protect the development of asthma. Traditionally, the adaptive immune system has been described as Th1 dominant or Th2 dominant, and asthma is associated with the Th2 phenotype (40). Furthermore, maternal Western diet exposure can reprogram the innate immunity of the offspring by altering the levels of aryl hydrocarbon receptor ligands, inducing a tolerant macrophage phenotype, which is associated with metabolic liver disease rather than a typical immune deficiency (98). These findings collectively suggest that during pregnancy and lactation, the maternal SCFA signals lay the foundation for the establishment of the offspring’s immune capacity.

Regarding intestinal health, butyrate attenuates inflammation through diverse mechanisms, including HDAC inhibition and activation of G-protein coupled receptors, primarily GPR109A and GPR41, and to a lesser extent GPR43. These mechanisms inhibit NF-κB activation and upregulate PPAR-*γ*, thereby promoting intestinal immune tolerance, increasing Treg populations (19, 33, 46, 99–101). Notably, butyrate-mediated induction of tolerogenic dendritic cells requires the combined action of both HDAC inhibition and GPR109A signaling (99). Building upon these molecular mechanisms, evidence indicates that maternal nutritional programming exerts long-term effects on offspring intestinal function (102, 103). In addition, butyrate has been shown to optimize microbial composition by inhibiting pathogenic bacteria while simultaneously promoting the proliferation of beneficial commensals (104, 105). Such effects collectively reinforce the intestinal barrier and contribute to the establishment of a resilient gut ecosystem that persists into later life.

With respect to body weight programming, clinical studies have revealed that offspring of mothers with higher SCFA levels exhibit more stable weight gain during infancy, reduced obesity risk, and more favorable body composition and metabolic profiles at birth (106, 107). Complementing these clinical observations, animal studies have confirmed that maternal SCFAs protect offspring against high-fat diet-induced obesity, with evidence demonstrating transmission via lactation (107). The underlying mechanisms identified in this context involve GPR41/GPR43 activation, which is associated with enhanced brown adipose tissue uncoupling protein 1 expression and increased energy expenditure (108). However, the potential involvement of HDAC inhibition in hypothalamic appetite regulation remains to be further investigated in the context of maternal SCFA programming (108). Beyond these well-characterized pathways, maternal SCFAs are also involved in the programming of offspring stem cell function. Emerging evidence suggests that SCFAs, functioning as inhibitors of HDACs, may activate the mTOR signaling pathway in progenitor stem cells by modulating the expression of stem cell markers such as leucine rich repeat containing G protein coupled receptor 5 (Lgr5) (109). These findings point to a potential crosstalk between the gut microbiota and neuronal or intestinal stem cells that fine-tunes their long-term functions, although further studies are needed to substantiate this hypothesis (109). The systemic programming effects of maternal SCFAs on offspring are summarized in Table 1.

**Table 1:** Summary of key evidence for maternal SCFA-mediated offspring programming.

Study design/model	Maternal exposure or SCFA-related factor	Main offspring findings	Programming domain/proposed mechanism	Ref.
Mouse, mechanistic study	Maternal gut microbiota-derived SCFAs during pregnancy	Maternal SCFA signaling, improved offspring glucose tolerance and reduced obesity susceptibility	Metabolic programming via embryonic GPR41/GPR43 signaling	Kimura et al. (38)
Mouse study	Maternal fiber deprivation with reduced SCFA-producing microbiota	Offspring showed altered gut microbiota, persistent low-grade inflammation, and increased susceptibility to obesity	Maternal low-fiber diet disrupts microbiota-SCFA axis and programs adverse metabolic outcomes	Zou et al. (80)
Multi-center prospective cohort	Antenatal depressive symptoms associated with reduced maternal butyrate-producing bacteria	Maternal antenatal depression at any trimester adversely affected infant neurodevelopmental outcomes	Neurodevelopment via maternal gut dysbiosis, reduced SCFA production, and inflammatory activation	Zhou et al. (90)
Prospective birth cohort	Infant gut microbiota composition shaped by maternal and perinatal factors	Gut microbiota composition at 2.5 months associated with infant temperament traits at 6 months	Neurodevelopment via early gut-brain axis signaling and potential SCFA-mediated pathways	Aatsinki et al. (91)
Mouse model of maternal obesity	Maternal high-fiber diet	High-fiber intake ameliorated offspring cognitive and social dysfunction, restored gut microbial balance	The cognitive and social dysfunction associated with maternal obesity is caused by the alteration of the intestinal microbiota in the offspring.	Liu et al. (92)
Mouse study	Maternal high-fiber diet during pregnancy and lactation	Increased differentiation of regulatory T cells in offspring	Immune programming through SCFA-mediated promotion of Tregs development	Nakajima et al. (93)
Human pregnancy cohort	Maternal fecal SCFA levels during pregnancy	Maternal SCFA profiles were associated with offspring asthma and allergic outcomes	Maternal SCFA status may influence allergic and immune risk in offspring	Lee-Sarwar et al. (96)
Prospective birth cohort	Infant diet (yogurt, fish, fruit, vegetables) associated with increased fecal butyrate levels	Children with highest fecal butyrate/propionate (≥95th percentile) had reduced atopic sensitization, asthma, food allergy, and allergic rhinitis by age 6	Immune tolerance via SCFA-promoted gut barrier integrity and regulatory T cell induction	Roduit et al. (97)
Pig model	Maternal short-chain fructo-oligosaccharide supplementation	Increased offspring intestinal butyrate concentration, goblet cell number, cytokine secretion, and vaccine response	Intestinal and immune programming; translational support from a large-animal model	Le Bourgot et al. (103)
Human observational study	Maternal and child SCFA profiles	SCFAs were associated with maternal and child anthropometric measurements	Supports a link between maternal SCFAs and offspring body weight/body composition programming	Szczuko et al. (106)
Mouse study	Maternal helminth infection altering microbiota and SCFA production	Offspring were protected against high-fat-diet-induced obesity	Body weight programming associated with maternal microbiota-SCFA remodeling	Su et al. (108)
Mechanistic study	Maternal gut microbiota and SCFA-related signaling	Maternal microbial environment influenced stem cell function in offspring	Stem cell programming involving microbiota-dependent metabolic and epigenetic regulation	Dang et al. (109)

SCFA, short-chain fatty acid; GPR41, G protein-coupled receptor 41; GPR43, G protein-coupled receptor 43; Tregs, regulatory T cells.

Although most studies support the protective effects of maternal dietary fiber on offspring health, some observational studies have reported inconsistent or even contradictory findings. For example, Abrahamsson et al. (110) found that reduced gut microbiota diversity during infancy (particularly regarding Bacteroidetes and Proteobacteria) was associated with an increased risk of atopic eczema, but no significant association was observed for Bifidobacteria or Clostridia. In contrast, Zhang et al. (111) using a herbivorous rodent model, reported that a maternal low-fiber diet unexpectedly promoted lean mass growth, improved glucose tolerance, and protected against high-fat diet-induced obesity in offspring. This conclusion cannot be directly applied to humans or omnivorous animals. Instead, it suggests that in cross-species transformation studies, species-specific digestive physiology and energy metabolism strategies must be taken into account. The heterogeneity underlying these inconsistent conclusions may arise from differences in population or species genetics and gut structure, baseline dietary composition, the types of samples used for SCFA detection, which reflect distinct metabolic windows, as well as variations in follow-up duration. Therefore, more high-quality human studies are needed to further confirm the beneficial effects of maternal high-fiber diet on offspring health.

## 4 Therapeutic perspectives

Fetal SCFA levels depend on maternal SCFAs transported across the placenta. After birth, breastfeeding becomes the main source of SCFAs for infants. During pregnancy, maternal SCFA cross the placental barrier, thereby exerting a fundamental programmed effect on the metabolism and immune development of the offspring (38, 57, 58, 93). Therefore, the time window for dietary fiber supplementation should cover the entire pregnancy and lactation period. Based on the review of our previous literature, the supplementation of SCFAs can be achieved through methods such as optimizing dietary fiber and probiotics.

Dietary fiber intervention represents the safest and most physiologically relevant approach. Although there are many benefits, excessive intake of dietary fiber may cause gastrointestinal discomfort. Currently, no safe upper limit (UL) for dietary fiber intake during pregnancy has been established. Accordingly, the U. S. Institute of Medicine sets the Adequate Intake at 28 g/day during pregnancy and 29 g/day during lactation, based on a criterion of 14 g per 1,000 kcal of energy intake, during the entire pregnancy and lactation periods, dietary fiber can be supplemented at this dosage (112). The Chinese guidelines for the management of gestational diabetes mellitus (2024) recommend a daily dietary fiber intake of 25–30 g for pregnant women with GDM (113). Given the absence of a defined UL, pregnant women are advised to maintain fiber intake within the recommended range to avoid potential gastrointestinal discomfort and impaired mineral absorption. Pregnant women should be encouraged to obtain fiber from diverse whole food sources such as whole grains, legumes, fruits, and vegetables (114, 115). Mechanistically, high-fiber diets enrich butyrate-producing bacteria such as Lachnospiraceae and elevate maternal butyrate levels, thereby attenuating placental inflammation through improved gut barrier function and reduced lipopolysaccharide translocation (25). These advantages support dietary fiber optimization as the first-line intervention during pregnancy.

Probiotic and prebiotic supplementation, by contrast, requires a more individualized risk benefit assessment. Maternal probiotic administration may increase Bifidobacterium abundance and acetate levels in mothers and breastfed infants (25), yet clinical efficacy remains inconsistent. The large SPRING trial demonstrated that a mixture of *Lactobacillus rhamnosus* combined with Bifi-dobacterium animalis subspecies lactis failed to prevent gestational diabetes in overweight and obese women (116). The results of another randomized double-blind clinical trial indicated that the intervention measures of using fish oil and/or probiotics during pregnancy seemed both safe and tolerable, but they did not have any benefits in reducing the risk of GDM or improving glucose metabolism in overweight and obese women (117). With respect to safety, commonly used strains of Lactobacillus and Bifidobacterium are generally well tolerated in healthy pregnant women, but the risk of systemic infection may rise in those with compromised immune function, short bowel syndrome, indwelling central venous catheters, or structural heart valve disease (118). In such cases, probiotic use should be supervised by a physician, and strains with higher invasive potential, such as Saccharomyces boulardii, should be avoided. Prebiotics including GOS, FOS and inulin may selectively stimulate SCFA-producing bacteria, but methodological factors such as source, degree of polymerization, and formulation remain inadequately characterized in current trials (119).

Given the substantial inter-individual variability in microbiome composition and dietary responses (120), a tiered precision nutrition strategy is warranted. Whole food dietary fiber should serve as the universally recommended foundation, supported by its established safety and consensus guideline endorsement. Probiotic or prebiotic supplementation may be considered as an adjunctive option for selected high-risk women, provided that contraindications related to immunocompromised states are carefully screened. Direct SCFA supplementation should remain restricted to research settings pending adequate human safety data. Future large-scale, rigorously designed clinical studies are still required to determine the long-term impacts, identify the most effective formulations, and understand how different populations varying by ethnicity, dietary habits, and metabolic profiles may respond.

### 4.1 Limitations of current research

Several limitations of the current literature should be noted. Most available studies are cross-sectional or based on small single-center cohorts, with limited longitudinal follow-up across pregnancy and few randomized controlled trials assessing microbiota or SCFA-targeted interventions. Methodological heterogeneity is also considerable, as differences in microbiota profiling techniques, SCFA quantification platforms, and pre-analytical procedures often hinder cross-study comparison. In addition, much of the mechanistic evidence is derived from rodent models, whose gestational physiology, placental structure, and microbial composition differ from those of humans, so extrapolation to clinical settings should be made with caution. Confounding factors such as maternal diet, body mass index, antibiotic or probiotic use, delivery mode, breastfeeding, and ethnic background are inconsistently controlled across studies. Future progress will depend on large, longitudinal, and ethnically diverse cohorts with harmonized methodology and more rigorous adjustment for confounding variables.

## 5 Conclusion and future perspectives

Maternal dietary fiber intake shapes offspring long-term health through gut microbiota-derived SCFAs. These microbial metabolites act via G-protein coupled receptor signaling and histone deacetylase inhibition to drive epigenetic reprogramming in developing tissues, influencing glucose metabolism, immune tolerance, neurodevelopment, intestinal barrier function, and adiposity. Studies in animals and humans have shown that supplementing dietary fiber can reduce these risks, and adequate dietary fiber supplementation during pregnancy is a safe and cost-effective public health strategy.

Nevertheless, several challenges hinder clinical translation. Individual variation in microbiome composition affects SCFA production capacity, and the optimal fiber type, dose, timing, and duration of supplementation remain poorly defined. Most human evidence is observational in nature, and randomized controlled trials with long-term offspring follow-up are scarce. Future priorities include well-designed trials incorporating microbiome stratification, standardized dietary interventions, and multi-omics profiling of mother-infant dyads.

It should also be acknowledged that most mechanistic evidence derives from rodent models. Species-specific differences in gestation length, placental architecture, gut microbial ecology, and SCFA receptor expression may limit direct extrapolation to humans. Translation into clinical practice therefore requires validation through prospective human cohort studies and randomized trials.
